# Supplementary material for: Risk of negative birth experience in trial of labor after cesarean delivery: A population-based cohort study
Source: PLoS One. 2020 Mar 6;15(3):e0229304. doi: 10.1371/journal.pone.0229304 (PMC7060072; doi:10.1371/journal.pone.0229304)
Supplement: S1 Table — (DOCX) [file pone.0229304.s001.docx]

**Supplementary tables**

**Table S1.** **Maternal characteristics by planned mode of delivery in 2^nd^ birth**

| **Characteristics 2^nd^ pregnancy** | **Planned mode of delivery in 2^nd^ birth (n= 808)** | | | | |
| --- | --- | --- | --- | --- | --- |
|  | **ERCD^a^** | | **TOLAC^b^** | |  |
|  |  |  |  |  | p-value |
| (n, %) | 251 | 31.1 | 557 | 68.9 |  |
| **Demographics** (Mean ±SD) |  |  |  |  |  |
| Age | 33.3 | 4.9 | 31.3 | 4.3 | <0.0001 |
| Height | 165.6 | 6.8 | 165.3 | 6.4 | 0.53 |
| BMI | 25.6 | 5.2 | 25.6 | 5.1 | 0.96 |
| **Social** (n, %) |  |  |  |  |  |
| Cohabiting | 240 | 95.6 | 535 | 96.1 | 0.77 |
| Smoker in early pregnancy | 5 | 2.0 | 19 | 3.4 | 0.22 |
| Alcohol Audit >6 | 1 | 0.4 | 2 | 0.4 | 0.95 |
| **Health** (n, %) |  |  |  |  |  |
| Received care for mental health issues | 15 | 6.0 | 31 | 5.6 | 0.07 |
| Self-assessed health at early pregnancy |  |  |  |  | 0.18 |
| *Very poor or poor* | 3 | 1.2 | 13 | 2.3 |  |
| *Neither poor or good* | 14 | 5.6 | 22 | 4.0 |  |
| *Good or very good* | 201 | 80.1 | 470 | 84.4 |  |
| *Missing or don´t know* | 33 | 13.2 | 52 | 9.3 |  |
| **Education** (n, %) |  |  |  |  | 0.008 |
| *≤9 years basic education* | 7 | 2.8 | 38 | 6.8 |  |
| *Secondary school education* | 70 | 27.9 | 197 | 35.4 |  |
| *University and college education* | 138 | 55.0 | 257 | 46.1 |  |
| *Missing or unknown* | 36 | 14.3 | 66 | 11.7 |  |
| **Birth experience 1^st^ birth** |  |  |  |  |  |
| *(Mean ±SD)* | 7.6 | 2.3 | 7.5 | 2.3 | 0.57 |
| *(Median and IQR)* | 8.0 | 3.0 | 8.0 | 3.0 |  |
| *Missing* | 78 | 31.1 | 215 | 38.6 |  |
| **Fear of childbirth in 2^nd^ pregnancy** (n, %) | 97 | 38.7 | 135 | 24.2 | <0.0001 |
| **Gestational age in weeks** (Mean ±SD) | 38.6 | 0.7 | 39.6 | 1.3 | <0.0001 |

^a^ERCD, elective repeat cesarean delivery; ^b^TOLAC, trial of labor after cesarean.
