# Supplementary material for: Risk of negative birth experience in trial of labor after cesarean delivery: A population-based cohort study
Source: PLoS One. 2020 Mar 6;15(3):e0229304. doi: 10.1371/journal.pone.0229304 (PMC7060072; doi:10.1371/journal.pone.0229304)
Supplement: S3 Table — (DOCX) [file pone.0229304.s003.docx]

**Table S3. Odds of negative birth experience by planned mode of delivery in 2^nd^ birth, logistic regression**

|  | **Negative birth experience 2^nd^ birth** | | | | | | | |
| --- | --- | --- | --- | --- | --- | --- | --- | --- |
| **Mode of delivery in 2^nd^ birth^b^** | **Crude** | | | | **Model 1^a^** | | **Model 2^a^** | |
|  | n | % | OR | 95% CI | aOR | 95% CI | aOR | 95% CI |
| **ERCD** | 8 | 3.2 | Reference | | Reference | | Reference | |
| **TOLAC** | 75 | 13.5 | 4.7 | 2.2- 10.0 | 4.5 | 1.9-10.7 | 3.1 | 1.0-9.4 |

^a^Adjustment in Model 1: maternal age, height, BMI, cohabiting, education, self-assessed health in 2^nd^ pregnancy. Model 2: same as in Model 1 and fear of childbirth in 2^nd^ pregnancy, birth experience after 1^st^ birth and mode of delivery in 1^st^ birth (elective vs unplanned CD). ^b^ERCD (elective repeat cesarean delivery), TOLAC (trial of labor after cesarean).
