# Supplementary material for: Risk of negative birth experience in trial of labor after cesarean delivery: A population-based cohort study
Source: PLoS One. 2020 Mar 6;15(3):e0229304. doi: 10.1371/journal.pone.0229304 (PMC7060072; doi:10.1371/journal.pone.0229304)
Supplement: S4 Table — (DOCX) [file pone.0229304.s004.docx]

**Table S4.** **Maternal characteristics by registered birth experience in 2^nd^ birth**

| **Characteristics 2^nd^ pregnancy**  (n= 931) | **Birth experience in 2^nd^ delivery** | | | |  |
| --- | --- | --- | --- | --- | --- |
|  | **Non-missing** | | **Missing** | |  |
|  |  |  |  |  | p-value |
| (n, %) | 808 | 86.8 | 123 | 13.2 |  |
| **Demographics** (Mean ±SD) |  |  |  |  |  |
| Age | 31.9 | 4.6 | 31.9 | 5.0 | 0.98 |
| Height | 165.4 | 6.5 | 164.6 | 7.0 | 0.20 |
| BMI | 25.6 | 5.1 | 25.7 | 5.2 | 0.85 |
| **Social** (n, %) |  |  |  |  |  |
| Cohabiting | 775 | 95.9 | 114 | 92.7 | 0.11 |
| Smoker in early pregnancy | 24 | 3.0 | 4 | 3.3 | 0.77 |
| Alcohol Audit >6 | 3 | 0.4 | 0 | 0 | 0.79 |
| **Health** (n, %) |  |  |  |  |  |
| Received care for mental health issues | 46 | 5.7 | 6 | 4.9 | 0.60 |
| Self-assessed health at early pregnancy |  |  |  |  | 0.43 |
| *Very poor or poor* | 16 | 2.0 | 1 | 0.8 |  |
| *Neither poor or good* | 36 | 4.5 | 9 | 7.3 |  |
| *Good or very good* | 671 | 83.0 | 99 | 80.5 |  |
| *Missing or don´t know* | 85 | 10.5 | 14 | 11.4 |  |
| **Education** (n, %) |  |  |  |  | 0.16 |
| *≤9 years basic education* | 45 | 5.6 | 11 | 8.9 |  |
| *Secondary school education* | 267 | 33.0 | 46 | 37.4 |  |
| *University and college education* | 395 | 48.9 | 48 | 39.0 |  |
| *Missing or don´t know** | 101 | 12.5 | 18 | 14.6 |  |
| **Birth experience 1^st^ birth** (n=587) |  |  |  |  |  |
| *(Mean ±SD)* | 7.5 | 2.3 | 7.7 | 2.4 | 0.63 |
| *(Median and IQR)* | 8.0 | 3.0 | 8.0 | 3.0 |  |
| *Missing* | 293 | 36.3 | 53 | 43.1 |  |
| **Fear of childbirth in 2^nd^ pregnancy** (n, %) | 232 | 28.7 | 32 | 26.0 | 0.41 |
| **Gestational age in weeks** (Mean ±SD) | 39.3 | 1.2 | 39.6 | 1.4 | 0.03 |
| **Mode of delivery in 2^nd^ birth^a^** (n, %) |  |  |  |  | 0.56 |
| *ERCD* | 251 | 31.1 | 36 | 29.3 |  |
| *VBAC* | 388 | 48.0 | 56 | 45.5 |  |
| *URCD* | 169 | 20.9 | 31 | 25.2 |  |

^a^ERCD (elective repeat cesarean delivery), VBAC (vaginal birth after cesarean), URCD (unplanned repeat cesarean delivery)
