# Supplementary material for: Risk of negative birth experience in trial of labor after cesarean delivery: A population-based cohort study
Source: PLoS One. 2020 Mar 6;15(3):e0229304. doi: 10.1371/journal.pone.0229304 (PMC7060072; doi:10.1371/journal.pone.0229304)
Supplement: Attachment — Submitted filename: Plos_One_birth_experience.docx [file pone.0229304.s005.docx]

**Reviewing : Plos One**

**« Risk of negative birth experience in trial of labor after cesarean delivery : a population-based cohort study.**

General comment:

The article shows data from a population-based cohort of women registered in the Swedish Pregnancy register. The topic of this study is of interest. As described by the authors, few publications focus on women's experience in second birth among women with a first cesarean. I found the manuscript well written overall.

Abstract

The abstract is clear. Minor comments: you may detail the number of women and frequencies in each categories for the exposure in the results section( n(%) for ERCD , URCD, VBAC). I would prefer using the terms of ‘odds’ instead of ‘risk’ when saying ‘URCD had five-fold …etc’.

Introduction

Very clear, thank you.

Methods

Please be more precise, if possible, when describing how women usually report their experience: is the scoring is self-administered or is the question asked by / in presence of the midwife? Are the answers anonymous? It could be a point of discussion: women may be intimidated to answer the question honestly in the presence of the caregiver tending to bias the responses toward a positive experience. Is the question about childbirth’s experience standardized across maternity units?

What do you mean by ‘organizational factors’ that could have an effect on the rate of reporting birth experience? Was the mean birth experience in these maternity units with birth experience response rate of less than 80% different from those with higher response rate ? Why choosing the cut off of 80% for exclusion?

Please consider rephrasing the sentences explaining the cut-off of 5 for defining negative birth experience. Here is a suggestion (line 130-131): “Previous studies … […]. The tenth percentile of the distribution of birth experience was 5 in our study. Thereby, to find women …etc”.

Do you have any information wether the decision of TOLAC or ERCD was shared with the women or decided only by the medical team? Many studies dealing with birth experience demonstrate the importance of shared-decision making process with the women; and that feeling of not having the choice was associated with lower satisfaction and experience, whatever the outcome.

Do you also have information on why ERCD was performed instead of TOLAC ? Is there clinical factors that systematically contra-indicate TOLAC and are those standardized across hospitals? Was the rate of TOLAC homogeneous across hospitals?

Statistical analysis

Before performing Student t test and linear regression, have you checked that hypothesizing a normal distribution seemed correct? Please consider justifying that somewhere.

Results

The Table 1 is not very clear and in line with your method section. As your exposure variable is 1) ECRD ; 2) unplanned repeated CD ; 3) VBAC, I recommend not to compare ERCD with TOLAC but to compare ERCD with 2) and 3) (two-by-two), as described in the method. It will also be more in line with the way you analysed them in the multivariable models. The rate of TOLAC may just be detailed in the text. Perhaps the differences in women’s characteristics between ERCD and TOLAC might be added as supplemental data ?

Line 178: the step-wise method for adjustment should be introduce in the method section and not in the results.

Table 2: it’s not very clear how you selected the confounders for mode l1 and model 2. Please better explain that in the method section.

Table 4: I’m not sure to clearly understand the table. What is the category of reference for the strata ‘unplanned CD in 1^st^ birth, 2^nd^ birth’? As far as I know, stratifying the results imply that the analyses of the effect of exposure on outcome are performed separately in each strata of a confounder (here elective or unplanned CD in first birth). Shouldn't the reference in stratum 2 be ERCD (but among women with 1^st^ unplanned CD) as well?

Line 198-9: I’m not sure you can conclude so strongly of a higher risk of negative second birth experience for women with unplanned CD in 1^st^ birth given the very small number of women and the very wide confidence interval. Perhaps moderate the sentence with ‘it seems that …’?

Table S1: it may be interesting to describe difference in hospital characteristics among women with and without missing data on childbirth experience (information as private maternity status and university status).

Discussion

Line 18-19: same comment as above.

Line 240-242: Couldn’t this assumption be easily verified?: were the scores of birth experience lower in hospitals were the scoring rate was lower than 80%?

Line 249: it’s not clear on which studies you referred to: what kind of intervention programs do you mean? We understand it later in the section but detailed it briefly here for a better understanding.

As commented before, I suggest that you discuss more about the importance of share decision making process and women’s involvement in this. It may be an element of explanation as to why the experience is high whatever the outcome of delivery.

You also introduce the importance of postpartum counselling in the conclusion. It’s an important element of discussion that, in my view, merit to be mention in the discussion section.
