# Supplementary material for: Risk of negative birth experience in trial of labor after cesarean delivery: A population-based cohort study
Source: PLoS One. 2020 Mar 6;15(3):e0229304. doi: 10.1371/journal.pone.0229304 (PMC7060072; doi:10.1371/journal.pone.0229304)
Supplement: Attachment — Submitted filename: Response to Reviewers.docx [file pone.0229304.s006.docx]

Dear Editor Dr Guillaume Ducarme,

Thank you for the careful review and comments on our manuscript entitled: ” **Risk of negative birth experience in trial of labor after cesarean delivery: a population-based cohort study**”, manuscript ID: **PONE-D-19-19552**.

We have revised our manuscript according to the comments by the Editor and Reviewers. The revised manuscript has been read and approved by all authors. Changes in the manuscript are indicated in the submitted version (‘track changes’). Our responses to the Editor’s and Reviewers’ comments are provided below in blue and italics.

Journal Requirements:

1. When submitting your revision, we need you to address these additional requirements.
   Please ensure that your manuscript meets PLOS ONE's style requirements, including those for file naming. The PLOS ONE style templates can be found at
   http://www.journals.plos.org/plosone/s/file?id=wjVg/PLOSOne_formatting_sample_main_body.pdf and <http://www.journals.plos.org/plosone/s/file?id=ba62/PLOSOne_formatting_sample_title_authors_affiliations.pdf>

We have revised the manuscript according to the style requirements of PLOS ONE. Please see submitted material.

2. We note that you have indicated that data from this study are available upon request. PLOS only allows data to be available upon request if there are legal or ethical restrictions on sharing data publicly. For information on unacceptable data access restrictions, please see http://journals.plos.org/plosone/s/data-availability#loc-unacceptable-data-access-restrictions.
In your revised cover letter, please address the following prompts:
a) If there are ethical or legal restrictions on sharing a de-identified data set, please explain them in detail (e.g., data contain potentially identifying or sensitive patient information) and who has imposed them (e.g., an ethics committee). Please also provide contact information for a data access committee, ethics committee, or other institutional body to which data requests may be sent.

Individual patient data from this study is not available on request due to legal aspects as data contains sensitive patient information. These restrictions are imposed by Swedish law. Hence, we are not able to provide a minimal anonymized data set. Request for data on an aggregated level may be sent to Olof Stephansson at the Clinical Epidemiology Division, Department of Medicine, Solna, Karolinska University Hospital and Institutet, Stockholm, Sweden, [olof.stephansson@ki.se](mailto:olof.stephansson@ki.se).

The following statement of data availability is true and correct:

*The data belong to "Graviditetsregistret" in Sweden, which does not permit data-sharing. Interested researchers who meet the criteria for access to confidential information may request data from info@graviditetsregistret.se. The researchers behind this study has requested data from Graviditetsregistret without any privileges. Request for data on an aggregated level may be sent to Olof Stephansson at the Clinical Epidemiology Division, Department of Medicine, Solna, Karolinska University Hospital and Institutet, Stockholm, Sweden, olof.stephansson@ki.se.*

b) If there are no restrictions, please upload the minimal anonymized data set necessary to replicate your study findings as either Supporting Information files or to a stable, public repository and provide us with the relevant URLs, DOIs, or accession numbers. Please see http://www.bmj.com/content/340/bmj.c181.long for guidelines on how to de-identify and prepare clinical data for publication. For a list of acceptable repositories, please see http://journals.plos.org/plosone/s/data-availability#loc-recommended-repositories.
We will update your Data Availability statement on your behalf to reflect the information you provide.

Please see above.

3. In ethics statement in the manuscript and in the online submission form, please provide additional information about the database used in your retrospective study. Specifically, please ensure that you have discussed whether all data were fully anonymized before you accessed them and/or whether the IRB or ethics committee waived the requirement for informed consent. If patients provided informed written consent to have their data used in research, please include this information.

Thank you for this important comment. We have now included the following paragraph in the Methods section (page 8, paragraph 4, lines 175-178):

*In this study, there were no requirement of informed consent from the women studied. The results are presented on an aggregated level and all data was fully anonymized in the research database. The regional ethical committee at Karolinska Institutet, Stockholm, Sweden approved the study (No 2017/2385-31/5 and No 2018/601-32).*

4. Your ethics statement must appear in the Methods section of your manuscript. If your ethics statement is written in any section besides the Methods, please move it to the Methods section and delete it from any other section. Please also ensure that your ethics statement is included in your manuscript, as the ethics section of your online submission will not be published alongside your manuscript.

Please see answer above (#3).

**Reviewing: Plos One**

**« Risk of negative birth experience in trial of labor after cesarean delivery: a population-based cohort study.**

ACADEMIC EDITOR: This article aims to investigate the birth experience related to the mode of de delivery after a previous cesarean section using data from a population-based cohort of women registered in the Swedish Pregnancy register. The topic of this study is of interest, whereas there are only few data in the literature. The article is well written and provide relevant information. But, some major revision should be done.

First, as noted by the two reviewers, the authors state that they included patients with a first cesarean and eligible for TOLAC. However, they don’t give any information in the methods section about what they consider being eligible for TOLAC. The authors should also provide details on the parameters which have leaded to the decision of TOLAC or ERCD.

Thank you for this important comment! We have included in both the Methods and Discussion section information about eligibility and decisions on delivery mode.

Method section (page 6, paragraph 1, lines 113-115): *Women were classified as eligible for TOLAC when the presentation was cephalic and there was no placenta previa or other medical contraindication for trial of labor.*

Discussion section (page 15, paragraph 1, lines 300-304):
*In Sweden, women with one previous cesarean delivery are recommended to undergo TOLAC if there are no contraindications for vaginal delivery. Women discuss mode of delivery with the antenatal midwife and she is encouraged to a trial of labor. If the woman is hesitant about trial of labor, she will be referred to an obstetrician.*

Second, as noted by Reviewer 1, another important point is that it would be relevant to compare the birth experience regarding the intent mode of delivery, not only the actual mode of delivery, i.e. TOLAC vs ERCD, not only VBAC vs ERCD vs URCD.

We agree with the Reviewer that the intent mode of delivery is important. The overall birth experience in the TOLAC group was 7.9 compared with 8.8 in the ERCD group. When stratified by mode of delivery, birth experience was 8.0 in the VBAC group and 7.6 in the URCD group. This result showed that the intention of mode makes a difference in birth experience, as well as the actual mode of delivery among the TOLAC group. In the revised Supplementary tables, we include the comparison between TOLAC and ERCD. We also include in the manuscript (Methods and Results sections) that we also made the comparison between ERCD and intended delivery mode (TOLAC).

Third, as noted by reviewer 2, major revision should be done to precise some results.

**Reviewer #1:**

This article aims to investigate the birth experience related to the mode of de delivery after a previous cesarean section.
It’s an interesting point whereas there are only few data in the literature. The article is well written and provide relevant information.

Here are several comments:

In the abstract, the authors state that they included patients with a first cesarean and eligible for TOLAC. However, they don’t give any information in the methods section about what they consider being eligible for TOLAC. Indeed not every patients with a previous cesarean section are eligible for TOLAC, depending on different surgical and obstetrical parameters.
The authors should provide details on the parameters which have leaded to the decision of TOLAC or ERCD. Did the patients have always the choice? In this kind of study, choice of the patients is a key-point since we can hypothesize that the birth experience might be better when the patients could actually choose the mode of delivery.

Please see comment above.

We have included in both the Methods and Discussion sections information about eligibility and decisions on delivery mode.

Method section (page 6, paragraph 2, lines 113-115): *Women were classified as eligible for TOLAC when the presentation was cephalic and there was no placenta previa or other medical contraindication for trial of labor.*

Discussion section (page 15, paragraph 1, lines 300-304):
*In Sweden, women with one previous cesarean delivery are recommended to undergo TOLAC if there are no contraindications for vaginal delivery. Women discuss mode of delivery with the antenatal midwife and she is encouraged to a trial of labor. If the woman is hesitant about trial of labor, she will be referred to an obstetrician.*

Another important point is that it would be relevant to compare the birth experience regarding the intent mode of delivery, not only the actual mode of delivery, i.e. TOLAC vs ERCD, not only VBAC vs ERCD vs URCD. Indeed, at the time we counsel patients, we don’t know how TOLAC will work…

Same as above.
We agree with the Reviewer that the intent mode of delivery is important. The overall birth experience in the TOLAC group was 7.9 compared with 8.8 in the ERCD group. When stratified by mode of delivery, birth experience was 8.0 in the VBAC group and 7.6 in the URCD group. This result showed that the intention of mode makes a difference in birth experience, as well as the actual mode of delivery among the TOLAC group. In the revised Supplementary tables, we include the comparison between TOLAC and ERCD. We also include in the manuscript (Methods and Results sections) sentences that we also made the comparison between ERCD and intended delivery mode (TOLAC).

VAS is a very poor tool to assess a complex feeling as birth experience. Even if the authors acknowledge limits of VAS in the discussion section, it should be more pointed out.

Thank you. We have added some more comments about VAS and how the question was put in the discussion part (page 14, paragraph 1, lines 271-278).

*Additionally, the question about birth experience is not standardized and non-anonymous, as it is asked by the midwife responsible for the postnatal care. Women may be intimidated or hesitant to answer the question honestly in the presence of a midwife with the tendency to bias the response toward a positive experience. There is also a risk that when asked about experience shortly after the birth the women might be affected by the so called halo-effect. Evidence shows that women rate their experience more positive shortly after birth compared with ratings later (5). However, this halo effect most likely affects all women regardless of mode of delivery..*

The figures (flow-chart and histograms) are of poor quality.

We have now improved the quality in Figures 1 and 2.

**Reviewer #2:**

General comment:

The article shows data from a population-based cohort of women registered in the Swedish Pregnancy register. The topic of this study is of interest. As described by the authors, few publications focus on women's experience in second birth among women with a first cesarean. I found the manuscript well written overall.

Thank you!

Abstract

The abstract is clear. Minor comments: you may detail the number of women and frequencies in each categories for the exposure in the results section( n(%) for ERCD , URCD, VBAC). I would prefer using the terms of ‘odds’ instead of ‘risk’ when saying ‘URCD had five-fold …etc’.

We have now in the Results section included number and rates of each delivery mode. Thereto we have changed risk to odds overall in the revised manuscript.

Introduction

Very clear, thank you.

Methods

Please be more precise, if possible, when describing how women usually report their experience: is the scoring is self-administered or is the question asked by / in presence of the midwife? Are the answers anonymous? It could be a point of discussion: women may be intimidated to answer the question honestly in the presence of the caregiver tending to bias the responses toward a positive experience. Is the question about childbirth’s experience standardized across maternity units?

We have included the following in the Methods section (page 5, paragraph 2, lines 99-103):

*The midwife responsible for the woman´s postnatal care asks the question about birth experience. It is asked either as an oral question or through a questionnaire, varying at different maternity units. The individual VAS scores are entered in the electronic medical records by the responsible midwife and forwarded to the Swedish Pregnancy Register.*

We have also included following in the Discussion section (page 14, paragraph 1, lines 271-278):

*Additionally, the question about birth experience is not standardized and non-anonymous, as it is asked by the midwife responsible for the postnatal care. Women may be intimidated or hesitant to answer the question honestly in the presence of a midwife with the tendency to bias the response toward a positive experience. There is also a risk that when asked about experience shortly after the birth the women might be affected by the so called halo-effect. Evidence shows that women rate their experience more positive shortly after birth compared with ratings later. However, this halo effect most likely affects all women regardless of mode of delivery.*

What do you mean by ‘organizational factors’ that could have an effect on the rate of reporting birth experience? Was the mean birth experience in these maternity units with birth experience response rate of less than 80% different from those with higher response rate? Why choosing the cut off of 80% for exclusion?

As for organizational factors, we refer to different approaches for evaluating childbirth experience with the VAS scale. The rationale for only including hospitals with a high response rate, arbitrary chosen as 80% or above, was to minimize potential biases. We have now included in the methods section following sentences (page 6, paragraph 2, lines 115-120):

*The preliminary analysis showed that some regions had a lower rate of reporting birth experience, possibly due to differences in the evaluation of the VAS scale as an instrument to measure birth experience, however response rates did increase over time. To diminish confounding by organizational factors, we chose arbitrarily to exclude women giving birth in hospitals with a birth experience response rate of less than 80% in 2017 (excluded hospitals n=23).*

We have now also included a sensitivity analysis that included all hospitals (except the Southeast Region hospitals). We found a similar mean and SD for the VAS score as when excluding hospitals with response rates below 80%:

- ERCD :	**8.8** SD 1.4 (only ≥80% responders: **8.8** SD 1.4)
- VBAC:	**7.9** SD 2.0 (only ≥80% responders: **8.0** SD 2.0)
- URCD:	**7.5** SD 2.3 (only ≥80% responders: **7.6** SD 2.1)

We have included these results in both the Methods (page 8, paragraph 3, lines 169-170) and the Results (page 12-13, paragraph 3, lines 237-240) sections:

*We performed a sensitivity analysis for mean VAS scores including all hospitals, except the Southeast region.*

*When including all hospitals, except the Southeast region with reverse interpretation of the VAS-scale, our sensitivity analysis showed little discrepancy from the results shown in Table 2 (mean VAS in ERCD 8.8, SD 1.4; VBAC 7.9, SD 2.0; URCD 7.5, SD 2.3).*

Please consider rephrasing the sentences explaining the cut-off of 5 for defining negative birth experience. Here is a suggestion (line 130-131): “Previous studies … […]. The tenth percentile of the distribution of birth experience was 5 in our study. Thereby, to find women …etc”.

We have rephrased the sentences as suggested by the Reviewer. (Methods section, page 7, paragraph 3, lines 143-146.)

*The tenth percentile of the distribution of birth experience was 5 in our study. Thereby, to find women scoring their birth experience as negative, we dichotomized the VAS score correspondingly and defined negative birth experience as VAS score ≤5.*

Do you have any information weather the decision of TOLAC or ERCD was shared with the women or decided only by the medical team? Many studies dealing with birth experience demonstrate the importance of shared-decision making process with the women; and that feeling of not having the choice was associated with lower satisfaction and experience, whatever the outcome.

In Sweden, women with one previous cesarean delivery are recommended to undergo TOLAC if there are no contraindications for vaginal delivery. Women who wish to discuss mode of delivery will be referred to an obstetrician in specialized antenatal care upon request.

See answer above and following sentences are added:

Method section: *Women were classified as eligible for TOLAC when the presentation was cephalic and there was no placenta previa or other contraindication for trial of labor.*

Discussion section:
*This difference in comparison with our results may be explained by the intervention program conducted in the study by Cleary Goldman et al. motivating women to perform a TOLAC (22). In Sweden, women with one previous cesarean delivery are recommended to undergo TOLAC if there are no contraindications for vaginal delivery. Women discuss mode of delivery with the antenatal midwife and she is encouraged to a trial of labor. If the woman is hesitant about a trial of labor, she will be referred to an obstetrician.*

Do you also have information on why ERCD was performed instead of TOLAC? Is there clinical factors that systematically contra-indicate TOLAC and are those standardized across hospitals? Was the rate of TOLAC homogeneous across hospitals?

Absolute contraindications for vaginal birth, including placenta previa, invasive placenta, transverse presentation among others, are standardized across Sweden. Relative contraindications for TOLAC or vaginal birth include two previous cesareans, non-isthmical previous incisions, breech presentation, grade 3 or 4 laceration with incontinence. However, in Sweden we tend to be generous allowing women with one previous cesarean having an ERCD upon request.

TOLAC rates in the included hospitals varied between 60 to 91%. We do not have specific information on the difference between hospitals. The difference may be explained by differences in maternal characteristics and variation in procedures. We have added in the Result section (page 9, paragraph 2, lines 182):

*TOLAC rates varied between 60-91% in the included hospitals.*

Statistical analysis

Before performing Student t test and linear regression, have you checked that hypothesizing a normal distribution seemed correct? Please consider justifying that somewhere.

We checked the normality assumption of linear regression through a qq plot, partly shown below:

We have included in the Methods section (page 6, paragraph 2, lines 161-163):

*Since the normality assumption of linear regression was not violated, we calculated the mean differences of birth experience (β-coefficient) through linear regression analysis.*

Results

The Table 1 is not very clear and in line with your method section. As your exposure variable is 1) ECRD ; 2) unplanned repeated CD ; 3) VBAC, I recommend not to compare ERCD with TOLAC but to compare ERCD with 2) and 3) (two-by-two), as described in the method. It will also be more in line with the way you analysed them in the multivariable models. The rate of TOLAC may just be detailed in the text. Perhaps the differences in women’s characteristics between ERCD and TOLAC might be added as supplemental data ?

We have now revised Table 1 according to the suggestions and we also include women´s characteristics under the Results section (page 9, paragraph 2-3, lines 184-189). We have also revised Supplementary Table comparing ERCD with TOLAC.

Line 178: the step-wise method for adjustment should be introduce in the method section and not in the results.

We have moved this to the Methods section (page 7, paragraph 4, lines 149-150), see below.

Table 2: it’s not very clear how you selected the confounders for mode l1 and model 2. Please better explain that in the method section.

Thank you for this comment. We have now added information on selection of confounders in the Methods section (page 7, paragraph 4, lines 148-158):

*Based on previous studies and clinical experience, we considered the following covariates as confounders. The covariates were adjusted for in a step-wise regression analysis. In Model 1 adjustments were made for maternal characteristics including maternal age at 2nd birth, body mass index (BMI), height and cohabiting at first antenatal visit in the 2nd pregnancy, education (≤9 years of basic education, secondary school, university or college education) and self-assessed health at early 2nd pregnancy (categorized from very bad to very good). In Model 2 we adjusted for the same covariates as in Model 1 and the women´s previous childbirth experiences such as fear of childbirth in 2^nd^ pregnancy (extra support from either midwife, obstetrician or psychologist), birth experience in 1^st^ birth (measured through VAS score) and additionally, mode of delivery in 1^st^ birth (elective or unplanned CD).*

Table 4: I’m not sure to clearly understand the table. What is the category of reference for the strata ‘unplanned CD in 1^st^ birth, 2^nd^ birth’? As far as I know, stratifying the results imply that the analyses of the effect of exposure on outcome are performed separately in each strata of a confounder (here elective or unplanned CD in first birth). Shouldn't the reference in stratum 2 be ERCD (but among women with 1^st^ unplanned CD) as well?

We have now categorized the women depending on the first delivery mode (elective or unplanned CD). We still wanted to compare all women with the reference category: women with two elective CDs.

Line 198-9: I’m not sure you can conclude so strongly of a higher risk of negative second birth experience for women with unplanned CD in 1^st^ birth given the very small number of women and the very wide confidence interval. Perhaps moderate the sentence with ‘it seems that …’?

We have now rephrased this sentence accordingly in the Result section (page 12, paragraph 2, lines 227-229):

*When further studying 1^st^ mode of delivery, it seems that women with a first elective cesarean and a second unplanned repeat CD…*

And in Discussion section (page 13, paragraph 2, lines 251-253):

*It seems that women who had an elective CD in first and unplanned CD in second delivery were at highest risk for having a negative birth experience.*

Table S1: it may be interesting to describe difference in hospital characteristics among women with and without missing data on childbirth experience (information as private maternity status and university status).

Table S1 describes the characteristics of all women in the hospitals that were included in the study.

We finally excluded 30 hospitals out of 42 included in the Swedish Pregnancy Register. These hospitals are spread out over Sweden and range between 1, 2 and 3 level hospitals. In general, maternity care is free of charge, financed by the public and carried out by public and private hospitals. There are no units lead by only midwifes. We have also now added this in the Methods section (page 5-6, paragraph 3, lines 106-110).

*All pregnant women are offered free maternity care in Sweden and the insurance system does not influence the availability of this care. More than 98% of pregnant women participate in the antenatal care system and more than 99% of all births take place in hospitals. The hospitals range between 1, 2 and 3 level hospitals and are mainly public (28). There are no units lead by only midwifes.*

Discussion

Line 18-19: same comment as above.

Line 240-242: Couldn’t this assumption be easily verified?: were the scores of birth experience lower in hospitals were the scoring rate was lower than 80%?

As described above, we have now added results for the VAS mean scores including all hospitals, except the ones from the Southeast Region with reversed interpretation of the scale (Results section, page 12-13, paragraph 3, lines 237-240).

*When including all hospitals in the Swedish Pregnancy Register, except the Southeast region with reverse interpretation of the VAS-scale, our sensitivity analysis showed little discrepancy from the results shown in Table 2 (mean VAS in ERCD 8.8, SD 1.4; VBAC 7.9, SD 2.0; URCD 7.5, SD 2.3).*

Line 249: it’s not clear on which studies you referred to: what kind of intervention programs do you mean? We understand it later in the section but detailed it briefly here for a better understanding.

We have no added following in the Discussion section under Interpretations (page 15, paragraph 2, lines 291-294):

*This study confirms that birth experience is associated with mode of delivery in women with a previous CD. This finding is in line with previous studies based on intervention programs with education of the women and decision-aids for counselling programs, aiming at improving birth experience.*

As commented before, I suggest that you discuss more about the importance of share decision making process and women’s involvement in this. It may be an element of explanation as to why the experience is high whatever the outcome of delivery.

Thank you for this comment. We have included under the Discussion section the following sentences (page 15, paragraph 2, lines 300-307):

*In Sweden, women with one previous cesarean delivery are recommended to undergo TOLAC if there are no contraindications for vaginal delivery. Women discuss mode of delivery with the antenatal midwife and she is encouraged to a trial of labor. If the woman is hesitant about trial of labor, she will be referred to an obstetrician. Obstetricians in Sweden tend to be generous allowing women with one previous cesarean having an ERCD upon request. Therefore, an explanation for general high birth experiences regardless of delivery mode could be the shared decision and good quality of care.*

You also introduce the importance of postpartum counselling in the conclusion. It’s an important element of discussion that, in my view, merit to be mention in the discussion section.

We have now included following at the end of the Discussion section (page 17, paragraph 2, lines 342-347):

*We suggest that women with a previous CD may benefit from an individual care plan throughout pregnancy and delivery. After childbirth, they ought to be screened for negative birth experience and offered counselling. We need to increase the awareness and skills among caregivers, with the aim of decreasing adverse outcomes and improve birth experience. More studies are needed in providing better support and maternity care.*
